# Moderate Intra-Abdominal Pressure Levels in Robot-Assisted Radical Prostatectomy Seem to Have No Negative Impact on Clinical Outcomes

**DOI:** 10.3390/jcm13051202

**Published:** 2024-02-20

**Authors:** Angelo Ippolito, Jan Mulier, Marta Hahn, Mike Wenzel, Philipp Mandel, Armin N. Flinspach, Katharina J. Wenger

**Affiliations:** 1 Department of Anaesthesiology, Intensive Care Medicine and Pain Therapy, University Hospital Frankfurt, Goethe-University Frankfurt, Theodor-Stern Kai 7, 60590 Frankfurt am Main, Germany; 2 Department of Anaesthesiology, Intensive Care and Reanimation, AZ Sint Jan Brugge, 8000 Bruges, Belgium; 3 Department of Anesthesiology, KULeuven, 3000 Leuven, Belgium; 4 Department of Anesthesiology, UGhent, 9000 Ghent, Belgium; 5 Department of Urology, University Hospital Frankfurt, Goethe-University Frankfurt, 60590 Frankfurt am Main, Germany; 6 Institute of Neuroradiology, University Hospital Frankfurt, Goethe-University Frankfurt, 60528 Frankfurt am Main, Germany

**Keywords:** robot-assisted radical prostatectomy, laparoscopy, pneumoperitoneum, intraabdominal pressure, postoperative inflammation, postoperative pain, dexamethasone

## Abstract

**Introduction:** Radical prostatectomy is increasingly performed laparoscopically with robot assistance (RALRP). RALRP, as with all laparoscopic procedures, requires a pneumoperitoneum, which might result in peritoneal inflammatory response reactions and postoperative pain. The aim of this retrospective single-centre study was to analyse the effects of a pneumoperitoneum during RARLP on clinical outcomes. **Methods:** All patients who underwent robot-guided prostatectomy in our clinic were included, with the exception of patients who were converted to open prostatectomy. C-reactive protein was used as a marker for the primary outcome, namely the postoperative inflammatory response. Intra-abdominal pressure (IAP) was evaluated as a potential factor influencing inflammation. In addition, the waist–hip ratio was used to estimate the amount of visceral adipose tissue, and the administration of dexamethasone was considered as a factor influencing inflammation. The Visual Analogue Scale (VAS) was used to determine postoperative pain. Patients were consecutively recruited between 1 September 2020 and 31 March 2022. **Results:** A total of 135 consecutive patients were included. The median waist–hip ratio was 0.55. The median duration of the pneumoperitoneum was 143 min. The median values of the average and maximum IAP values were 10 mmHg and 15 mmHg, respectively. The mean CRP of the first postoperative day was 6.2 mg/dL. The median VAS pain level decreased from 2 to 1 from the first to the third postoperative day. On the first postoperative day, 16 patients complained of shoulder pain. In addition, 134 patients were given some form of opioid pain treatment following surgery. **Conclusion:** We could not identify any relevant associations between the duration and IAP of the pneumoperitoneum and the indirect markers of inflammation or indicators of pain, or between the latter and the amount of visceral adipose tissue. In addition, we found no significant effect of the administration of dexamethasone on postoperative inflammation. The results point to a noninferior tolerability of moderate pressure during the procedure compared to the commonly utilised higher pressure, yet this must be confirmed in randomised controlled trials.

## 1. Introduction

Robot-assisted radical prostatectomy (RARP) is considered the current ‘gold standard’ in surgery for localised prostate cancer [1,2].

Like other laparoscopic procedures, it employs pneumoperitoneum, which is brought about by insufflating the abdominal cavity with a gas (commonly CO_2_), thereby providing the surgeon with the visibility necessary to perform the procedure. While the routine intra-abdominal pressure (IAP) maintained during the use of pneumoperitoneum is on average 12–15 mmHg, international guidelines recommend ‘the lowest intra-abdominal pressure allowing adequate exposure of the operative field rather than a routine pressure’ [3,4].

Several factors of the RARP procedure can cause adverse effects in comorbid patients. During the procedure, patients are most commonly placed in the steep Trendelenburg position [5]. The surgical positioning together with the insufflation of the abdominal cavity can affect cardiovascular and pulmonary functions [3,5,6,7,8,9,10,11,12,13]. Due to its absorption, CO_2_ decreases the peritoneal pH level. The lower pH increases the likelihood of an inflammatory response [14,15,16]. In addition, commonly used insufflation pressure levels of up to 15 mmHg can cause barotrauma to the peritoneal serosa, changing its integrity [17] and promoting inflammatory response [17]. Lastly, the amount of the patients’ visceral adipose tissue has been reported to increase the level of local inflammation [17,18,19]. Both inflammatory responses and adhesions caused by capnoperitoneum can result in increased pain levels [20]. It has been demonstrated in previous studies that low IAP is associated with less postoperative pain [10,15,21].

The primary endpoint of this retrospective clinical study was to analyse the clinical outcomes of patients with pneumoperitoneum during RARP procedures in terms of the inflammatory response. As a secondary objective, the effects of the administration of dexamethasone on postoperative inflammation and pain were studied.

## 2. Materials and Methods

### 2.1. Study Design

Consecutive patients admitted for RARP between 1 September 2020 and 31 March 2022 were included in this single-centre, retrospective study. All patients who underwent RARP in our clinic were included, with the exception of patients whose procedures were converted to open prostatectomy. The study was approved by the institutional review board (document number 2023-1177). Data were collected in accordance with the data collection and data protection guidelines of the institution. The investigators planned and designed this study in accordance with the recommendations of the Declaration of Helsinki.

### 2.2. Data Curation

Data describing patient characteristics, such as patient age during the surgical procedure, height, weight, and waist circumference, were recorded using a patient data management system (mobile anaesthesia documentation system ‘Sandman.MD’ by AppAtWork, hospital information system, ORBIS). Body mass index (BMI) was calculated using the height and weight. The waist–hip ratio of the patients was calculated by dividing the waist circumference by height and was used to estimate patients’ visceral fat [22].

Furthermore, the following data regarding the procedure itself were collected before and after the pneumoperitoneum procedure: end-tidal CO_2_ and O_2_, inhaled O_2_, ventilation, and CO_2_ production. The respiratory quotient was calculated through the ratio of end-tidal CO_2_ and the difference between the inhaled O_2_ and end-tidal O_2_. In addition, the duration of the pneumoperitoneum, the insufflation pressure and volume at the start of the pneumoperitoneum, the average and maximum intraabdominal pressure during the pneumoperitoneum, the total dose of neuromuscular blocking agents, and the positioning of the patient during the pneumoperitoneum procedure were recorded. A ratio was also calculated using the total dose of neuromuscular blocking agents and the duration of pneumoperitoneum in hours. Furthermore, an index was built using the product of IAP during pneumoperitoneum and the duration of pneumoperitoneum in minutes.

Moreover, the following data were collected with regard to the analgesic drugs administered: the intraoperative administration of anti-inflammatory drugs, the amounts of opioids used intraoperatively and postoperatively (absolute dosage and in morphine equivalents), and whether analgesic additives were used. Inflammation was determined by C-reactive protein levels. The IAP and the duration of pneumoperitoneum were analysed in terms of their correlation with postoperative inflammation, measured through C-reactive protein levels. Furthermore, the waist–hip ratio, as a surrogate for visceral adipose tissue content, was tested for an association with postoperative inflammation.

In order to quantify postoperative pain in the first three postoperative days, two methods were employed. Patients were asked to report if they felt shoulder pain at all, and if yes, whether the pain was located on the left- or right-hand side shoulder or both. In addition, patients were asked to report the level of pain according to the Visual Analogue Scale (VAS).

The C-reactive protein (CRP) levels of the first three postoperative days were recorded as indicators of inflammation and were extracted manually from the laboratory information management system LAURIS.

Several patients were administered dexamethasone as prophylaxis for postoperative nausea and vomiting (PONV). This was at the discretion of the treating physician after considering the risk factors for PONV. If indicated, the administration of dexamethasone and the dosage administered were documented to test for a relationship with postoperative inflammation.

### 2.3. Statistics

The statistical analysis of the collected data was performed using the Statistical Package for Social Sciences version 28 (SPSS, Chicago, IL, USA). The value of α was set to 0.05 according to convention and *p*-values less than 0.05 were considered as statistically significant. Additionally, *p*-values were reported without correction for multiple testing, enabling a thorough exploration of potential associations across various parameters.

The characteristics of the study population, RARP procedures, and the postoperative clinical outcomes were summarised using descriptive analysis and frequencies. Interval-scaled data were analysed for normal distribution comparing a histogram with the normal distribution curve and using the Kolmogorov–Smirnov test, which is suitable for study populations greater than 50 [23]. The mean and standard deviation (SD) were used to summarise interval-scaled variables with a normal distribution, while the median and interquartile range (IQR) were used to summarise interval-scaled variables lacking a normal distribution [24]. Parametric and nonparametric statistical tests were selected depending on the presence or absence of a normal distribution of the data. To assess the relationship between variables, Kendall’s Tau was employed due to its lower sensitivity to ties (cases where two or more observations have the same rank) compared to other nonparametric correlation coefficients, and because it does not assume a normal distribution of the variables. This measure was employed to analyse the relationship between clinical outcome factors, such as CRP, VAS, and postoperative opioid use, against the parameters of PP, which were the duration of PP and the average and maximum IAP. The dependent variables in these analyses were the parameters of PP. The Kruskal–Wallis test, chosen for its ability to compare non-normally distributed variables between more than two groups, was used to analyse the association between shoulder pain and the parameters of PP. The dependent variables in this case were likewise the parameters of PP. Furthermore, the effect of the waist–hip ratio (substitute for visceral fat) on the postoperative inflammatory response (measured by CRP) was tested using Kendall’s Tau test, with the waist–hip ratio being the dependent variable. The association between the application of dexamethasone on the dependent variables of postoperative VAS pain level and shoulder pain were analysed using the Mann–Whitney U test and Pearson’s chi-squared test, respectively.

Descriptive statistics and linear regression analyses were conducted in both groups to determine the potential influence of BMI, duration, volume, and waist circumference on postoperative pain.

## 3. Results

### 3.1. Characteristics of the Study Population

Demographic and anthropometric characteristics of the study population (*n* = 135 male patients) are listed in Table 1. The median age of the population at the time of surgery was 67 years (IQR 10; range 39–79). The BMI of the population had a median value of 26.87 kg/m^2^ (IQR 5.31; range 19.88–40.96), with none of the patients being underweight, 38 patients (28.1%) being normal weight, 62 patients (45.9%) being overweight, and 35 patients (25.9%) being obese.

The waist–hip ratio was calculated using the waist circumference and height of the patients, and it had a median of 0.55 (IQR 0.09; range 0.44–0.78).

### 3.2. Characteristics of the RALRP Procedures Performed

RALP characteristics were identical among the study population and were performed as follows:

- Number of trocars: 4;
- Trocar sizes: four 8 mm trocars (three trocars for the robotic arms and one for the camera/extraction), one 5 mm trocar (for assistant or suction), and one 12 mm trocar (for assistant or insufflation);
- Insufflation: high-flow, low-pressure pneumoperitoneum; standard IAP 10 mmHg, temporary increase to 15 mmHg during the manipulation of the prostatic plexus for 1–3 min, reduction to 5 mmHg during the lymphadenectomy in the final 20 min of the surgery.

Among the total of 135 patients included in this analysis, 132 patients (97.8%) were placed in the steep Trendelenburg position, while 3 patients (2.2%) were placed in the lateral position. The properties of pneumoperitoneum utilised in the RALRP procedures of this study population are summarised in Table 2.

The total dose of neuromuscular blocking agents administered intraoperatively had a median of 80 mg rocuronium (IQR 35; range 40–150). The ratio of the dose of neuromuscular blocking agents per unit of time had a median of 30.97 mg/h (IQR 15.29; range 16.09–72).

A total of 129 patients (95.6%) were administered the anti-inflammatory drug metamizole intraoperatively. Among these patients, 123 (95.3%) were administered with 1 g of metamizole, while 6 (4.7%) were administered with 2 g of metamizole.

Among the study population, 12 patients (8.9%) were given dexamethasone for PONV prophylaxis. Among these patients, 2 patients (16.67%) received 4 mg of dexamethasone, while 10 patients (83.33%) received 8 mg of dexamethasone.

### 3.3. Postoperative Clinical Outcomes

**Figure 1:**
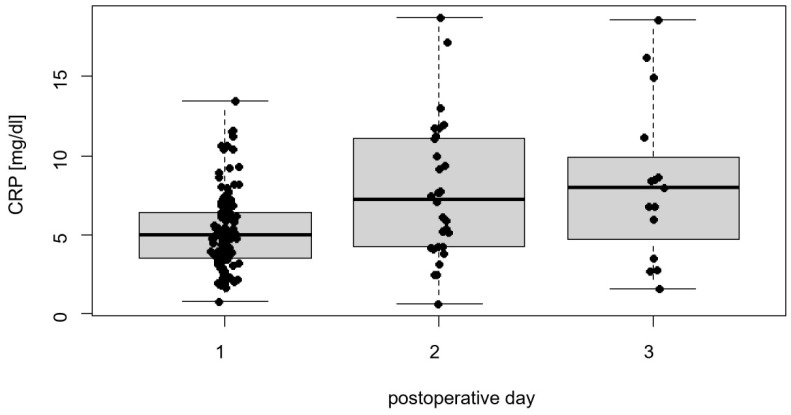
Distribution of CRP values on the first three postoperative days.

The primary endpoint of this study was the postoperative outcome of patients after pneumoperitoneum during RARP, with a focus on the inflammatory response, as assessed by CRP levels. Notably, 39 patients (28.89%) had a recorded CRP value on the first postoperative day, with a mean CRP of 6.20 ± 2.43 mg/dL (range 1.92–13.49, Figure 1). The CRP levels of the second and third postoperative days were recorded for 30 (22.22%) and 15 patients (11.11%), respectively, and they had median values of 7.26 mg/dL (IQR 6.89; range 0.62–18.74) and 7.96 mg/dL (IQR 7.68; range 1.54–18.59), respectively.

The postoperative use of opioids was reported in morphine equivalents. All but one patient had valid data on the postoperative administration of opioids. Among these patients, a median value of 4.2 morphine equivalents (IQR 4.9; range 0–24.92) was reported, disregarding tramadol administrations. A median of 24.2 morphine equivalents (IQR 4.9; range 20–44.92) was reported when tramadol was considered.

On the first postoperative day, 129 patients (95.56%) reported a median VAS pain level of 2 (IQR 1; range 0–8). Among these patients, 113 patients (87.6%) reported no shoulder pain, while 7, 7, and 2 patients reported pain in the left, right, and both shoulders, respectively.

On the second postoperative day, 130 patients (96.3%) reported a median VAS pain level of 2 (IQR 1; range 0–5). Notably, 118 (90.77%) patients reported no shoulder pain, while 4 and 7 patients reported pain in the left and right shoulders, respectively. One patient reported pain in both shoulders.

On the third postoperative day, 131 patients (97.04%) reported a median VAS pain level of 1 (IQR 2; range 0–5). Of those, 129 patients (98.5%) reported no shoulder pain. One patient reported pain in the right shoulder, and one reported pain in both shoulders.

### 3.4. Effects of Capnoperitoneum on the Clinical Outcome

The CRP level on the first postoperative day was tested against the duration of capnoperitoneum, the average IAP, and the maximum IAP separately using Kendall’s Tau test. The duration of capnoperitoneum (PP) showed a very weak positive association with a correlation coefficient (cc) of 0.033, which was not statistically significant (*p* = 0.771). The average IAP and the maximum IAP of PP showed very weak (cc = −0.01) and weak (cc = −0.153) negative correlations, respectively, that were not statistically significant (*p* = 0.939 and 0.221, respectively).

**Figure 2:**
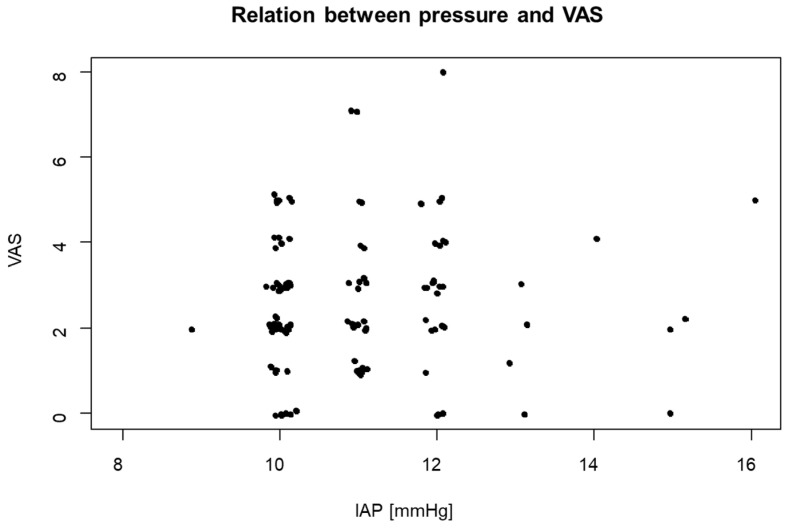
VAS values on the first postoperative day in relation to IAP.

The VAS pain level on the first postoperative day was also analysed in terms of its correlation with the duration of PP, the average IAP, and the maximum IAP using Kendall’s Tau test. The test resulted in very weak positive correlations with cc values of 0.007, 0.03, and 0.058, respectively, which were all statistically insignificant (*p* = 0.915, 0.682, and 0.431, respectively, Figure 2).

Shoulder pain was analysed for a significant association with the parameters of PP using the Kruskal–Wallis test. All three parameters, i.e., the duration of PP, average IAP, and maximum IAP, showed no significant differences in the association of mean values among the four categories of shoulder pain on the first postoperative day (*p* = 0.539, 0.073, and 0.241, respectively).

Postoperative opioid use (with and without the inclusion of tramadol use) was tested against the duration of PP, the average IAP, and the maximum IAP using Kendall’s Tau test. All three variables showed very weak correlations with cc values of −0.029, 0.004, and 0.085, respectively. All three results were not statistically significant (*p* = 0.63, 0.949, and 0.22, respectively).

### 3.5. Effects of Visceral Fat on the Inflammatory Response

The marker for visceral fat, the waist–hip ratio, was tested against the CRP levels of the first three postoperative days, in order to investigate the influence of the degree of visceral fat on inflammation. All three Kendall’s Tau tests were statistically insignificant (*p* = 0.62, 0.843, and 0.891, respectively), with cc values of 0.061, −0.028, and 0.03, respectively.

### 3.6. Effects of Dexamethasone on the Clinical Outcome

The administration of dexamethasone was tested against the VAS pain level on the first postoperative day and opioid use (with and without the inclusion of tramadol) using the Mann–Whitney U test. All three tests resulted in statistically insignificant results (*p* = 0.800, 0.916, and 0.916, respectively). The presence or absence of shoulder pain was analysed against the administration of dexamethasone using Pearson’s chi-squared test. The result was not statistically significant (*p* = 0.674). Nonetheless, all of the 16 patients who complained of some form of shoulder pain were in the cohort that did not receive dexamethasone.

As all 12 patients who were administered dexamethasone lacked valid CRP levels on postoperative day one, a Student’s *t*-test could not be performed.

## 4. Discussion

This retrospective single-centre study analysed the effects of the use of capnoperitoneum during RARP procedures on the postoperative clinical outcome, more specifically on inflammation (measured by postoperative CRP levels) and postoperative pain (represented by VAS pain levels, the presence or absence of shoulder pain, and the use of opioids).

It seems that the moderate pressure levels used (average IAP 10 mmHg) in this retrospective cohort were well tolerated. Previous studies prospectively stratified patients into groups with very low pressures and higher pressures. A recent, randomised clinical trial assessed the impact of the IAP on the postoperative quality of recovery, measured with the QoR-15 questionnaire, and stratified patients undergoing RALRP into two IAP groups (low pressure = 7 mmHg vs. standard pressure = 12 mmHg) [10]. The results demonstrated several advantages of low IAP for a beneficial postoperative outcome, including higher QoR-15 scores, and significant improvements in physical and emotional well-being. Another randomised trial compared two patient groups of low (6 mmHg) and high (15 mmHg) IAP in terms of postoperative pain and use of analgesics and found significantly lower mean pain scores, maximum pain scores, shoulder pain, and groin pain in the low IAP group [11]. In a study by Christensen et al., a pressure value of 12 mmHg was compared to 15 mmHg, with the outcome of patients in the lower-pressure group not significantly differing from patients in the higher-pressure group [25]. The lack of statistically relevant differences between both groups could potentially be explained by the fact that a difference of 3 mmHg was too small to lead to any differences in the outcome.

While the expansion of the peritoneum leading to inflammation and the activation of the immune response has been demonstrated in animal studies [11], the results of the present retrospective study on human patients cannot corroborate these findings, as no relevant correlations between the inflammation marker CRP and the average and maximum IAP were observed [8]. It appears that the clinical surrogate markers for peritoneal inflammation do not correlate with the level of the IAP. One explanation for this negligible association could be the lower IAP utilised during the majority of the duration of the RALRP procedures performed on this study population and the standard IAP utilised in the majority of the study populations of the current literature. Several studies have reported that using a low-pressure pneumoperitoneum is not inferior to using a standard-pressure pneumoperitoneum and results in more favourable postoperative clinical outcomes [26,27]. Regardless, RARP procedures have been reported to have much more favourable results with regard to surgical stress and acute phase systemic response when compared to alternative radical prostatectomy procedures without robotic assistance [28].

Data regarding the effect of the duration of PP on postoperative inflammation and pain are scarce in the current state of research. The analysis of the data collected for this study showed that RARP procedures that lasted longer caused only slightly increased levels of postoperative CRP and pain levels, despite the results being statistically insignificant.

Even though the current literature states that visceral adipose tissue can act as a promoting factor for inflammatory responses, our study was unable to identify a statistically significant and consistent relationship between the two factors [17,18,19]. In addition, other factors and comorbidities, such as chronic infections or cardiovascular and metabolic diseases, may impact the occurrence and level of inflammation after prostatectomy.

Finally, as a second objective of this study, we investigated the possible effect of dexamethasone in reducing the inflammatory response and pain after RALRP procedures in order to assess the effect of anti-inflammatory agents administered after such procedures reported in previous studies [29,30,31]. Even though no statistically significant result was obtained, all patients who complained of shoulder pain after surgery belonged to the cohort that did not receive dexamethasone.

This study has several limitations. The study lacks randomisation, which may give rise to various forms of bias. As retrospective analyses are based on the data obtained and documented in patients’ charts in the past, the selection of patients and relevant data is solely the decision of the observer and may therefore skew actual outcomes. The lack of valid data on important variables, for example, the lack of CRP values on the first postoperative day among 71.11% of the population, resulted in the inability to perform certain statistical tests and created a bias, thereby causing the loss of valuable statistical information. Moreover, preoperative CRP values and comparison to the postoperative values could have been useful in investigating changes in intrinsically high CRP values in prostate cancer patients. Unfortunately, preoperative data were unavailable, and due to the small number of patients with postoperative CRP values, this evaluation would likely not have yielded reliable results.

## 5. Conclusions

In conclusion, in this single-centre, prospective clinical study, we could neither identify relevant associations between the duration and IAP of pneumoperitoneum and the postoperative CRP, pain, and use of opioids nor establish any association between the amount of visceral adipose tissue and postoperative inflammation and pain. In addition, we found no significant effect of the administration of dexamethasone on postoperative inflammation. It therefore remains unclear how the available information on low-to-moderate pressure levels may translate into clinical practice. The fact that moderate pressure levels are associated with certain patient benefits, including the avoidance of barotrauma to the peritoneal serosa and inflammation, may render a decrease in pressure a valid option during prostatectomy. Our results point to a good tolerability of the procedure when moderate pressure is used, but this must be investigated in randomised controlled trials in the future.

## Figures and Tables

**Table 1:** Demographic and anthropometric patient characteristics.

Parameter	
Age in years, median	67 (IQR 10; range 39–79)
Weight in kg, median	85 (IQR 18; range 61–125)
Height in m, mean ± SD	1.78 ± 0.06 m (range 1.60–1.96)
BMI in g/m^2^, median	26.87 (IQR 5.31; range 19.88–40.96)
Waist circumference in cm, median	97 (IQR 17.3; range 78–131)
Waist–hip ratio	0.55 (IQR 0.09; range 0.44–0.78)

**Table 2:** Properties of pneumoperitoneum (PP) utilised in the RALRP procedures.

Property of PP	Median
Duration	143 min (IQR 46; range 94–265)
Insufflation pressure at start	10 mmHg (IQR 1; range 7–17)
Insufflation volume at start	4.35 L (IQR 2.3, range 1.5–50.6)
Average IAP	10 mmHg (IQR 2; range 8–16)
Maximum IAP	15 mmHg (IQR 1; range 10–22)
Duration × average IAP	1529 mmHg min (IQR 570; range 972–3180)

## Data Availability

Raw data were generated at the University Hospital of Frankfurt. Derived data supporting the findings of this study are available from the corresponding author A.I. upon reasonable request.
